# A method for assessing links between objectively measured food store scores and store & neighborhood favorability

**DOI:** 10.1186/s12942-019-0195-7

**Published:** 2019-12-27

**Authors:** Richard C. Sadler, Ashley N. Sanders-Jackson, Josh Introne, Robyn Adams

**Affiliations:** 1 Division of Public Health, Michigan State University, 200 E 1st St Room 337, Flint, MI 48502 USA; 2 Department of Advertising and Public Relations, Michigan State University, East Lansing, USA; 3 Department of Media and Information, Michigan State University, East Lansing, USA; 4 Department of Advertising + Public Relations, Michigan State University, East Lansing, USA

## Abstract

Worldwide, interest in research on methods to define access to healthy food at the local level has grown, given its central connection to carrying out a healthy lifestyle. Within this research domain, papers have examined the spatial element of food access, or individual perceptions about the food environment. To date, however, no studies have provided a method for linking a validated, objective measure of the food environment with qualitative data on how people access healthy food in their community. In this study, we present a methodology for linking scores from a modified Nutrition Environment Measures Survey in Stores (conducted at every store in our study site of Flint, Michigan) with perceptions of the acceptability of food stores and shopping locations drawn from seven focus groups (n = 53). Spatial analysis revealed distinct patterns in visiting and avoidance of certain store types. Chain stores tended to be rated more highly, while stores in neighborhoods with more African-American or poor residents were rated less favorably and avoided more frequently. Notably, many people avoided shopping in their own neighborhoods; participants traveled an average of 3.38 miles to shop for groceries, and 60% bypassed their nearest grocery store when shopping. The utility of our work is threefold. First, we provide a methodology for linking perceived and objective definitions of food access among a small sample that could be replicated in cities across the globe. Second, we show links between perceptions of food access and objectively measured food store scores to uncover inequalities in access in our sample to illustrate potential connections. Third, we advocate for the use of such data in informing the development of a platforms that aim to make the process of accessing healthy food easier via non-food retail based interventions. Future work can replicate our methods to both uncover patterns in distinct food environments and aid in advocacy around how to best intervene in the food environment in various locales.

## Introduction

Food access and the study of so-called ‘food deserts’—or low-income areas where people have limited access to healthy food—have emerged as major inquiries within public health: while just 7 studies on food deserts were conducted prior to 1990, 12 were conducted in the 1990s, and 29 more were conducted in the 2000s [4]. The evolution of this work is of interest generally because limited access to affordable, healthy food has been linked to poor dietary habits [12, 13], which in turn can contribute to a range of negative health outcomes including heart disease [48], obesity [10], and diabetes [61]. This focus on food deserts is but one way of examining the issue of food access, but represents the increased interest in addressing the issue of food access, something that was once seen outside the purview of planners and policymakers [49].

At its most basic, research incorporating GIS in the study of food access frequently examines proximity to or density of major food store types (such as supermarkets) as the operationalization of ‘access’. The USDA’s former Food Desert Atlas, for example, previously designated food deserts based solely on the absence of a large grocery store or supermarket (combined with socioeconomic deprivation) [68]. Researchers have criticized such approaches, however, because they provide little or no insight into how people’s perceptions of their food environment align (or fail to align) with these simplistic measures [59].

To address such concerns, research has begun to link consumer and community food environments using methods that more realistically estimate the types of foods available in stores and communities. Even so, many studies continue to oversimplify either the consumer or community food environment side of the equation through poorly designed or non-existent store audits, and thus have not as-yet created methods for linking how people perceive the food environment with an objective measure of the food environment. Two primary gaps in food access research of interest in this paper include: (1) many objective food environment measures do not take into account the variability of healthy food availability from store to store; and (2) methods for linking spatially referenced perceptions of the food environment to these audits are not yet in common use.

Although little evidence has been gathered actually linking the products available in stores to eating behaviors, many major proposals to ameliorate food access-related issues have involved a food retail intervention such as the opening of a new grocery store [7, 51, 58, 73] or enrichment of existing stores with more healthy options [13]. This proposal is premised on the idea that not only ‘if you build it, they will come’ but that ‘they will change what they eat’. Such sentiment is not universally shared, however, because peoples’ use of the food environment is more complex than only seeking out healthy foods at major grocery stores (e.g. people shop at other types of stores and frequently buy food that is suboptimal for health). Researchers thus increasingly do not favor this simplistic policy proposal, and instead have favored alternative means of helping people access and consume healthy foods [7, 8, 46].

Since we know merely opening new stores is not sufficient for changing behavior, understanding perceptions of food access is also important. Indeed, in some studies, authors suggest perceptions to be a primary determinant in what foods are purchased [26, 27, 29]. Others are more apprehensive, finding small or no relationships between perception and diet, diet-related outcomes, or weight [20, 40]. Likewise, links between the actual food environment and peoples’ perception remain unclear; while some studies suggest they may be proxies for one another [43], others suggest a poor connection between the two [70].

Given the above, both understanding the composition of the food environment and how people use their food environments are important for creating methods to study the relationship between food access and diet, and thus for understanding how best to intervene to help people eat more healthy food. Additionally, food retail interventions require great capital investment, thus community development entities with a stake in social justice can find the cost to entry prohibitive. In the absence of models that emphasize alternative food systems, this gap leaves conventional food retailers (whose profit-driven models often don’t jibe with social need) as the primary agent shaping the food environment. Because of this struggle to ensure healthy food is distributed equitably, behavioral strategies (e.g. encouraging healthier eating through non-retail based interventions) that acknowledge the current state of the food system and capitalize on what is known about structural forces in the food system are necessary.

Our immediate objective is to combine perceptions about food shopping and self-reported shopping behavior with a food store survey to provide a method for exploring how people use their food environments. This offers an improvement on existing methods because we found no studies linking objective food environment data with food store perceptions. The methods used in this study will therefore be of interest widely to international audiences interested in linking objective and perceived food environments. Secondarily, this work will inform the creation of a food-system based sociotechnical platform that will connect people to the food system and yield better opportunities to impact diet. Without first understanding the nature of the food system as perceived by community residents, such an endeavor would be less likely to succeed. By combining objective data about the food system with impressions about the food system, we aim to create a platform that is responsive to the concerns of community residents.

Tools for objectively measuring the quality of the food environment are well-established, but do not always effectively integrate spatial measures. Because of the presumed effect of the food environment on diet and, therefore, health outcomes [44], better precision in measuring the food environment is necessary if researchers are going to continue to make explicit claims about such relationships.

At the consumer (or store) level, the Nutrition Environment Measures Survey (NEMS) (introduced in [18] is considered a high-quality option for measuring the quality, variety, and price of healthy foods at the store level because it is both valid and reliable [39]. Few studies have used validated tools like the NEMS at the community level, many instead using more simplistic food basket surveys incorporating only availability and occasionally price of goods [63, 67]. Minaker et al. [40] adapted the NEMS in Stores (NEMS-S) to incorporate some measures of the neighborhood environment by creating 1 km (km) buffers around the home location of research participants, but until Shaver et al. [62] the NEMS-S had not been appended to neighborhood-level characteristics. Thus drawing inference about the relationship between food access and community characteristics remained underdeveloped.

Additional food environment metrics also tend to focus on community-level environments. The validated Retail Food Environment Index (RFEI) measures the relative number of unhealthy vs. healthy food outlets in a community (typically aggregated to a census unit) [2]. The RFEI, however, does not provide in-depth information on the quality of products within ‘unhealthy’ vs. ‘healthy’ stores. RFEI has been used in tandem with GIS by linking RFEI values to activity spaces [21] or calculating distance buffers around locations and calculating average RFEI scores to capture a food environment [64]. In Franco et al. [15], the creation of a Health Food Availability Index for stores was similarly aggregated up to obtain an average neighborhood healthy food availability score. Work on geodemographic and population segmentation likewise aims to add depth to the understanding of the food environment, clustering neighborhoods or people according to demographics, proximity to/density of different food store types, attitudes about healthy eating, and approaches to obesity prevention [38, 71]. Additional work examined the community food environment through GIS analysis [3, 11, 16], but a common strain in much of this work is the absence of objective measures of the in-store (consumer) environment (such as the NEMS-S) in the creation of community-level measures of food access. That is, granular data about food available *in* stores isn’t represented in aggregate at *broader scales*.

To counter this lack of understanding, Kumar et al. [33] recommended linking objective measures like the NEMS-S to perceptions of food access, which could provide a better understanding of how well people ‘know’ healthy options available in their food environments (with respect to the items collected on the NEMS-S). Examination of Kumar’s subsequent work does not reveal a follow-up to this recommendation. Thus, we see a fruitful opportunity to build on Kumar et al.’s recommendation that exploratory/qualitative research on food access should be combined with objective measures like the NEMS-S to bridge perceptions of food access and quality among community residents.

## Methodology

### Study community

Flint, Michigan, is noteworthy both for its decimated food retail system and the public health crisis that engulfed residents since 2014 [22]. Disinvestment over many years created an environment where traditional food retail options were greatly diminished in quality: only one chain grocery store exists in a city of nearly 100,000 people, and a handful of independent grocers lag behind the quality, variety, and price of the chains that dominate the suburban fringe. This gap presumably causes many residents to drive outside the city to reach affordable, healthy food. Recent research quantified the gaps, signifying that poorer neighborhoods and neighborhoods with higher proportions of African-American residents had lower FFSS scores (and, therefore, poorer quality and variety of healthy foods) [62].

Earlier grocery-store related investments failed [58], though more recent initiatives such as a mobile market and further investments in a farmers’ market and mobile market [56, 57] have been sustained over longer periods. As well, a healthy corner store program rolled out by the state university extension helped several storeowners stock more healthy foods through subsidy of produce, coolers, and freezers (MSU [45]. It is not yet clear, however, whether these smaller-scale interventions will yield a positive effect on Flint residents. Recognizing the need for a broad spectrum of interventions, our research team and community partners have proposed another option for addressing these ongoing challenges through the creation of a socio-technical platform called ‘Flint Eats’. This technology would send spatially and temporally targeted healthy eating messages to platform users, and integrate information about these efforts and more into one place, making healthy choices more obvious in the food environment.

In this paper, we draw on two primary data sources to propose a methodology to link different aspects of food access. The first is a survey of perceptions of the acceptability of food stores and shopping locations drawn from seven focus groups (n = 53 people) conducted in the summer of 2016. The second is the Flint Food Store Survey (FFSS), a NEMS-S adapted objective measure of the food environment in Flint, Michigan, collected in September 2016 [62]. The latter survey provides a view of healthy food availability, quality, and variety in every store in and around our study site. The temporal concordance affords us the opportunity to draw comparisons between perceptions of food access and objectively measured food access at the same stores and in the same neighborhoods. This is done in part by linking the mapped locations of food shopping (or avoidance) with food store scores from the FFSS.

### Focus groups

In addition to standard university IRB approval, our focus groups were approved by the Healthy Flint Research Coordinating Center’s Community Ethics Review Board [23], which was created to address imbalances in academic-community interactions. Participants were recruited via community e-mail distribution lists, at farmers’ markets and other community events, and most importantly through word-of-mouth and community groups themselves. Our intention was not to obtain a representative sample of the city, but rather one that overrepresented people from the northern side of Flint, where food access is particularly problematic and where concern about inequity in the food system is more pervasive.

Because of long-standing concerns about helicopter research and unequal access to the benefits from research [24], we specifically sought to minimize practices that would be perceived as reinforcing inequitable methods for collecting data. As such, we collected limited demographic data. The participants were fairly educated: 35% had a Bachelor’s degree or higher, and another 52% had at least some college training. In terms of income, 45% made less than $30,000 a year, 30% made between $30,000 and $60,000, and 25% made over $60,000. We did not collect data on age, race, or gender, but observation of the group suggested that they were overwhelmingly older, African-American, and female.^1^

On the day of the focus group, participants arrived at either the city library (considered a ‘neutral’ location) or a local church (one group specifically selected this location, as it was more central to their neighborhood than the library). Participants were consented both verbally and in writing and asked to complete a short questionnaire about their eating habits, experiences living and shopping in Flint, and social media and technology use.

Once all participants completed the survey, and participated in an icebreaker question, they were asked to complete a personalized mapping exercise to circle where they primarily buy groceries, where they do not travel (or shop), and where they live. In small groups, they were then asked to share further information about where they do not go and what they purchase or do not purchase at particular stores on a common/shared map. The exercise elicited both a rich conversation about place in Flint and information about where and how people obtain food and make food-related decisions.

Hand-drawn map-based data was digitized into ArcGIS 10.3 (ESRI, Redlands, CA). Places where participants indicated purchasing food were assigned to the associated store, while places people avoided were classified based on the location and size of the circled areas as either a store or a neighborhood. The rate of participants reporting either shopping at or avoiding specific stores was enumerated to create a favorability rating (e.g. the number reporting a store as favorable/the total number of ratings). Stores were subsequently sized according to how many participants mentioned them and colored according to their favorability rating. Areas and stores participants avoided were placed on top of one another to generate a gradient of areas that people are more likely to avoid (the store avoidance and neighborhood avoidance variables explored below). The center point of circled neighborhoods was designated as an approximate home location to give an anonymized impression of where individuals lived.

### Food store survey

We also make use of a community-wide food store survey developed using the Nutrition Environment Measures Survey and deployed in September 2016. Surveyors collected information on the availability, price, and quality of dairy products (max observed = 17 pts), fruits & vegetables (max = 35 pts), proteins (max = 9 pts), grains (max = 27 pts), and lead-mitigating foods (max = 5 pts) at every store in and within two miles of the City of Flint, with a maximum possible store score of 93. The comprehensive assessment afforded the creation of store score surfaces (detailed in [62], whereby every location in the city was assigned food availability scores based on individual food categories and the overall store score.

We then combined impressions of area stores from the focus groups with the FFSS scores to determine links between perceived store quality and objectively measured quality, variety, and price, using both summed scores and individual category scores as noted above.

### Socioeconomic characteristics

Lastly, to measure the relationship between neighborhood socioeconomic status and food store quality as reported by our participants, we used census block group-level variables from the American Community Survey, including lone parenthood, poverty, unemployment, educational attainment, food stamp usage, and percent African-American population. The first four have been commonly used in calculating socioeconomic distress indices (as in [58]. Food stamp usage is considered important in the consideration of food shopping [14], while percent African-American is important as a contextual variable for race-related disinvestment in the study community [60].

### Distance to shopping

Using the estimated home location derived from the maps alongside the linked sites where our participants reported shopping, we were able to compute approximate distances to stores using Network Analyst in ArcGIS (ESRI). Both the actual stores visited and the nearest stores (of any kind, and including only chain grocery stores) were calculated.

## Results

**Fig. 1:**
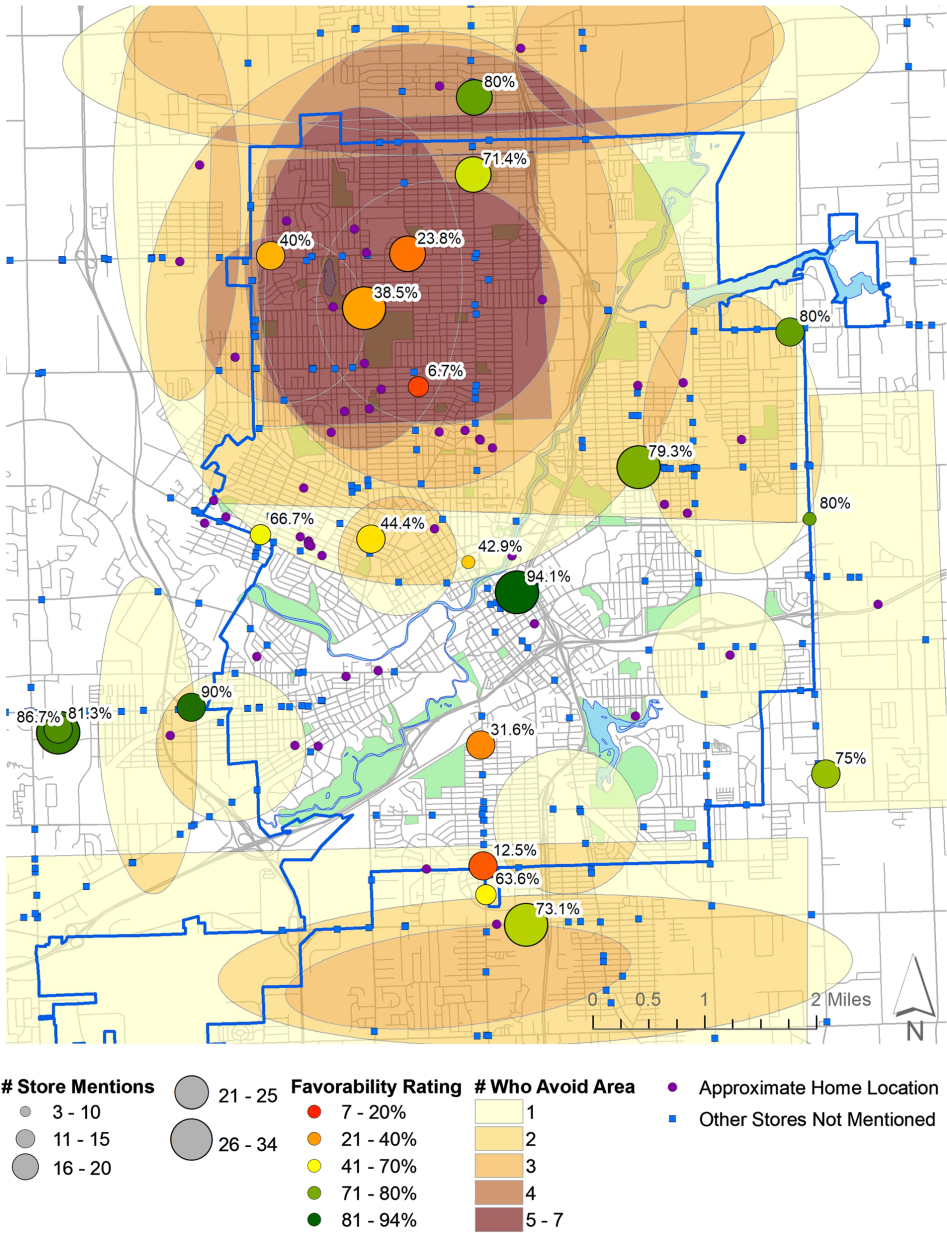
Map of Flint showing focus group participant-provided stores shopped at, overall store favorability rating, areas avoided, and approximate home locations

Figure 1 shows a visual depiction of the variables used in Table 1 below. Areas that our participants avoid are shown in shades of yellow-to-brown (based on the number of people who reported avoiding those neighborhoods). The average favorability score of each store is shown along a red-green scale, with greener colors representing a higher favorability rating. The size of the circles is based on the number of people who mentioned each store. The number of people who avoided specific stores is not shown on this map but is included in Table 1 below. The purple dots signify the approximate home locations of our participants.

**Table 1:** Average NEMS-S scores by stores visited, reported favorability, store avoidance, and neighborhood avoidance

	Stores		Store favorability		Store avoidance		Neighborhood avoidance		
	Rated	Not rated	High (≥ 70%)	Low (< 70%)	Low (< 10)	High (≥ 10)	None (0)	Some (1–3)	Many (≥ 4)
n	22	244	11	11	15	7	6	10	6
Fruit score (max = 19)	14.77	2.74	16.55	13.00	15.93	12.29	17.17	15.45	11.33
Vegetable score (max = 16)	12.45	1.34	13.27	11.64	12.67	12.00	12.67	12.36	12.67
Fruit score + vegetable score (max = 35)	27.23	4.08	29.82	24.64	28.60	24.29	29.83	27.82	24.00
Alternative fruit score (sum) (max = 19)	15.32	1.25	18.00	12.64	17.00	11.71	17.83	15.91	12.00
Alternative vegetable score (sum) (max = 24)	19.32	1.45	22.00	16.64	21.00	15.71	20.33	20.73	16.17
Alternative fruit score + vegetable score (sum) (max = 43)	34.64	2.69	40.00	29.27	38.00	27.43	38.17	36.64	28.17
Alternative fruit score (direct) (max = 13)	10.73	0.90	12.55	8.91	11.80	8.43	12.50	11.27	8.00
Alternative vegetable score (direct) (max = 15)	12.73	0.91	14.36	11.09	13.67	10.71	13.67	13.00	11.50
Alternative fruit score + vegetable score (direct) (max = 28)	23.45	1.81	26.91	20.00	25.47	19.14	26.17	24.27	19.50
Dairy score (max = 17)	11.82	3.55	14.09	9.55	13.67	7.86	12.67	13.36	8.17
Meat score (max = 9)	8.34	5.22	8.34	8.34	8.22	8.61	8.17	8.34	8.42
Grain score (max = 27)	19.77	7.80	21.82	17.73	20.87	17.43	17.50	21.09	20.83
Lead-mitigating food score (max = 5)	4.91	3.73	4.82	5.00	4.87	5.00	5.00	4.82	4.67
Store score (max = 91)	72.52	24.37	79.80	65.25	76.88	63.18	73.17	76.34	66.08
Alternative store score (Sum) (max = 97)	79.48	23.00	89.07	69.89	85.62	66.32	81.50	84.25	70.25
Alternative store score (direct) (max = 84)	68.30	22.12	75.98	60.61	73.08	58.04	69.50	71.89	61.58

Of the 22 stores that were rated for favorability, 6 were considered conventional chain stores (average FFSS score = 83.3), 4 were discount chain stores (avg. FFSS = 74.1), 10 were independent grocers (avg. FFSS = 67.7), and 1 was a farmers’ market (FFSS = 54.25). As might be expected based on store size and affordances, chain stores scored highest in favorability (81.3%), followed by discount stores (67.7%) and independents (46.5%). The farmers’ market was an outlier, rating 94.1% favorable despite its smaller size. Given these differences in store favorability, the spatial distribution of different types of stores likely influences food access.

Notably, Fig. 1 also shows the overlap in places where participants live, places they avoid, and poor favorability scores, primarily in the northern part of the city (suggesting that our participants who live in northern Flint often do not shop there). Using the data from the FFSS, we test the assumption that poorly regarded stores in neighborhoods participants avoid will have consistently lower scores.

Table 1 illustrates the relationship between objectively-derived food store scores (FFSS scores, from the NEMS-S) and perceptions of food stores and the food environment among our participants. Columns include the favorability rating and avoidance level of individual stores, as well as the level of avoidance of certain areas (e.g. how many focus group participants noted that they avoided the area when shopping more generally). The FFSS scores are subdivided by category type and include two alternative scoring methods for fruits and vegetables [62]. As well, Table 2 illustrates the average socioeconomic conditions in the neighborhoods around each grouping of stores (e.g. by store favorability, store avoidance, and neighborhood avoidance).

**Table 2:** Neighborhood characteristics of stores visited, reported favorability, store avoidance, and neighborhood avoidance

	Stores		Store favorability		Store avoidance		Neighborhood avoidance		
	Rated	Not rated	High (≥ 70%)	Low (< 70%)	Low (< 10)	High (≥ 10)	None (0)	Some (1–3)	Many (≥ 4)
n	22	244	11	11	15	7	6	10	6
Lone parenthood (%)	0.30	0.31	0.28	0.33	0.28	0.36	0.21	0.29	0.41
Poverty (%)	0.35	0.35	0.31	0.38	0.32	0.40	0.25	0.36	0.42
Unemployment (%)	0.21	0.20	0.17	0.25	0.17	0.28	0.14	0.19	0.31
No high school Diploma (%)	0.29	0.25	0.25	0.33	0.27	0.34	0.18	0.33	0.33
Lone parenthood z-score	− 0.06	0.00	− 0.33	0.22	− 0.32	0.51	− 0.98	− 0.18	1.08
Poverty z-score	0.01	0.00	− 0.27	0.29	− 0.16	0.37	− 0.74	0.12	0.56
Unemployment z-score	0.04	− 0.01	− 0.36	0.43	− 0.33	0.83	− 0.72	− 0.19	1.16
No high school diploma z-score	0.28	− 0.03	0.00	0.56	0.10	0.66	− 0.54	0.58	0.60
Socioeconomic distress	0.27	− 0.04	− 0.97	1.50	− 0.71	2.37	− 2.97	0.33	3.40
Food stamp usage	0.38	0.37	0.35	0.40	0.35	0.43	0.27	0.40	0.44
African-American	0.46	0.41	0.31	0.62	0.33	0.75	0.28	0.34	0.85

In total, participants reported visiting or specifically avoiding 22 unique stores for their primary grocery shopping (almost entirely grocery stores or supermarkets), leaving 244 additional stores not rated by participants. Of the stores not visited, only 6 had scores high enough to be considered a grocery store (with an average FFSS score of 79), 226 scored lower than any store noted by participants (35 of those had a score of 0), and the average score of 24.0 was 21 points lower than the lowest scoring store rated by participants. Almost all of these 244 stores can be classified as liquor stores, gas stations, or dollar stores. They are excluded from analysis because our focus here is on the characteristics of stores based on their reported favorability, and because we asked only about stores our participants visited with regularity (and which therefore have a strong impact on their level of food access).

Of the visited stores, 11 had favorability ratings at or above 70%, while the other 11 were below 70%. Fifteen stores had low store avoidance scores (< 10 participants reported not visiting them), while 7 had high scores (10 or more participants avoided them). In terms of neighborhood avoidance, 6 stores were in neighborhoods that no one avoided, 10 were in neighborhoods that some avoided (1–3), and 6 were in neighborhoods that many avoided (4 +).

### Store favorability

FFSS scores of all types were higher in highly favorable stores than less favorable stores (see Table 1). The overall FFSS score of favorable stores was 79.80, compared to 65.25 for less favorable stores. The only scores that were broadly the same across both categories were meat scores (both 8.34) and lead-mitigating food scores (4.82 vs. 5.00).

Stores with high favorability scores also existed in neighborhoods with lower rates of all measures of socioeconomic distress and food stamp usage (see Table 2). For instance, the unemployment rate in neighborhoods around stores with high favorability was 17%, while it was 25% in neighborhoods around stores with low favorability. These stores were also in places that had overwhelmingly fewer African-American residents (only 31% of the population) when compared to the neighborhoods surrounding stores with lower favorability scores (where they comprised 62% of the population).

Certain stores were outliers with respect to the general relationship between FFSS score and store favorability. Most notably, while the farmers’ market had only an FFSS score of 54.25, it was very highly rated in terms of favorability (94.1%). Conversely, two local stores had very low favorability ratings (12.5% and 23.8%), despite having fairly good FFSS scores of 71.75 and 76.25.

### Store avoidance

Patterns of store avoidance mirrored favorability scores. Again in every category except meat scores and lead-mitigating food scores, stores that participants avoided less had much higher FFSS scores than stores that were avoided more. The disparity between these stores with respect to neighborhood-level food stamp usage and percent African-American residents was even higher. 35% of residents in neighborhoods of stores with no avoidance used food stamps compared to 43% in neighborhoods of stores with high avoidance scores. Stores with low avoidance scores also resided in neighborhoods where the population was only 33% African-American. This is compared to stores with high avoidance scores, where the neighborhood surrounding them was 75% African-American.

### Neighborhood avoidance

We also examined neighborhood-level avoidance more generally, to understand places that our participants might ‘write off’ completely when considering where to shop. Some of the biggest disparities are seen in this category. The unemployment rate in neighborhoods participants avoid is more than double the rate in neighborhoods no one avoids (31% vs. 14%). Socioeconomic distress is more than six standard deviations different in high avoidance vs. no avoidance neighborhoods (3.40 vs. − 2.97, where 0.00 is the mean distress score). And neighborhoods our participants avoid are 85% African-American, compared to 28% African-American in neighborhoods that are not avoided.

### Average distance to shopping

On average, participants in our study traveled 3.38 miles to shop for groceries. Only 11% shop within 1 mile of their homes, despite the fact that the average distance to any grocery store from participants’ homes was only 0.79 miles, and the average distance to a chain grocery store was only 2.75 miles. Most participants (60%) travelled farther than the closest store to purchase food, further contextualizing how Flint residents bypass existing stores to reach other food sources because of perceived issues with food quality, and because car ownership makes such choices much easier [6].

## Discussion

The purpose of our research is to combine exploratory/qualitative research on food access with objective food access measures to understand perceptions of access and quality among a non-representative sample of community residents. Not only have we shown how our focus group participants bypass their nearest stores to reach healthy foods, we demonstrate that many stores being bypassed tend to have poorer quality foods as measured by the FFSS (as illustrated by the fact that the average score of the 244 non-rated stores was only 24.37, compared to 72.52 for the stores visited).

Perhaps not surprisingly, we illustrated that the attraction of large chain grocery stores may be drawing our participants away from smaller retailers in their neighborhoods (as in [34] and [66]. We suggest that this may be attributable to lower prices, larger variety, and higher quality in these stores, as supported by our observation that chain stores had higher average FFSS scores.

Our observation that the average distance traveled to grocery stores is 3.38 miles is somewhat smaller than that found in Kerr et al. [30] of 6.3 miles. This may be expected given that our study area is much smaller than the region studied that in paper (Atlanta), but is still instructive by highlighting how residents bypass available food sources. Conversely, our work contrasts with DiSantis et al. [9] entirely, who found most people in their sample were shopping within a mile of their home, and cited convenience as a primary driver of that shopping behavior. In contrast, Thornton et al. [66] discuss how younger people in their sample were willing to travel greater distances for food. These variegated results highlight the ongoing need for qualitative research examining why people shop the way they do.

We have also provided further evidence that poor quality stores are located in poorer neighborhoods and neighborhoods with higher concentrations of minority populations. This supports a range of literature indicating how such neighborhoods experience a deprivation amplification [37] or additional environmental injustice [25] due to their socioeconomic status. It also reflects work by Alkon et al. [1] that people living in poor communities employ a range of strategies to reach healthy foods.

We found that our focus group participants avoided neighborhoods of particular compositions more than the stores within them. Our participants were more likely to avoid neighborhoods entirely that were more heavily African-American, had higher poverty rates, and where the unemployment rate was higher. This is in contrast to Lee’s [35] work, which suggested African-American shoppers experienced more harassment at stores in predominately white neighborhoods, but accords with previous research where participants cited safety concerns of shopping in some neighborhoods [54].

### Limitations

We acknowledge some limitations in our data. While we collected the focus groups concurrently with the original food store survey, these data are at the time of publication 3 years old. They have been influencing policymaking around food retail provision, but are already becoming out-of-date. We should be sensitive to changes in the food retail environment that have occurred since then.

Using focus group-collected data to quantify perceptions of food access is problematic, and we caution what can be inferred from our work. We do not make claims of statistical significance, and reported store ratings should therefore be interpreted with this in mind. Rather, our intention was to illustrate how perceptions of food access and shopping behaviors among a small sample of Flint residents link with our objective food store survey.

Relatedly, we recruited focus group participants without the intention of being representative of the population. The northern side of the city of Flint has been of central discussion in local efforts to improve food access [41, 42, 55], and we therefore sought to recruit people from these areas who would be interested in an intervention to ameliorate some of these issues. Our maps, therefore, do not suggest we are making claims representative of the population overall (nor even of the northern side of Flint). Rather, the method of participatory mapping and linking to objectively measured food environments is itself one of the major methodological contributions. Indeed, this method illustrates an approach for gathering new dimensions of food access among a specific subset of the population (without making claims of generalizability) that could be replicated in a wide variety of contexts across the globe.

### Policy implications

This work has important public health implications by creating a method for further understanding residents’ avoidance behavior of stores near their homes; namely, our participants perceived the quality of food stores in undesirable neighborhoods to be poor, even though an objective assessment suggested healthy foods were available at some stores. The Office of Disease Prevention and Health Promotion (ODPHP, n.d.) suggests that health starts in an individual’s home, community, and neighborhood. Factors such as a person’s built environment influence how an individual will make decisions about where to access the foods that encourage healthy eating (ODPHP, n.d.). This work not only supports the inferences of public health theories such as the Social Determinants of Health, it also provides a methodology for improving the understanding about how more objective spatial tools can be used in combination with current assessments to understand the impact of the built environment on health. This is important, as some research in built environment and health is limited in that it only examines individual home infrastructures (e.g. housing quality) or social factors (e.g. crime and violence).

Furthermore, this work has policy implications by illustrating connections among where residents do or do not shop in their communities. By linking community members’ perceptions of food stores to objectively-derived measures from the FFSS, our results reveal that stores being passed by are of poorer quality than the stores ultimately being visited. This may explain in part why only 22 stores were noted by the 53 participants—many stores may simply not be worth shopping at for regular groceries. Thus, replicating this method via additional focus groups or surveys would be valuable for local governments who are interested in addressing gaps in the food environment through zoning policies. Such policies can help determine the designation and location of community gardens and food markets, the limitation of junk food sites like fast food restaurants and convenience stores, and the offering of incentives to local businesses to increase the access of quality foods within their stores [36, 50, 65].

Although changes can be made through local policy to improve access to healthy foods in communities like Flint, there is still a need for an accessible communication channel before and after these changes that gives community members access to information on where to get these foods. Our work and replicated studies elsewhere can therefore provide support in developing a tailored communication tool (in our case, such as the app noted above) that assists community members in making decisions on food shopping and healthy eating. Early communications research suggests that tailored communication is effective in enhancing message relevance [32]. Moreover, this research also suggests that health communication materials or tools that seek to make information more releveling to their target audience are more effective those that do not [32]. Previous research in tailored health communication stresses the importance of understanding individual differences as a way to address health concerns [52]. This current work offers a similar approach by using measures of community members’ perceptions and spatial analysis tools to provide a method that can help us understand why individuals make decisions about where they food shop. Information from this work can be integrated with current knowledge on tailored communication to develop a tool that helps individuals with improving their food shopping choices. Most fundamentally, the wide relevance of our methodology offers the opportunity for communities in the US and internationally to replicate our work and study links between perceived and objective food access, with the goal of becoming more responsive to potential non-retail based solutions.

## Data Availability

The datasets collected and analyzed as part of the current study are not available in original form due to confidentiality requirements, but anonymized data are available from the corresponding author upon request.

## Footnotes

1 While we were able to observe some participant characteristics, we did not ask these questions for specific reasons. Although the primary researcher on this paper has worked in Flint for many years, recruiting participants from a predominately African-American community with a history of experiencing structural racism always presents significant challenges. In general, African-Americans are more skeptical of participation in research [5, 17]. The Flint Water Crisis itself was associated with a significant decrease in trust of government [19], and this may impact how researchers from state institutions (such as our university) are perceived. Non-traditional methods of participant recruitment that focus on developing relationships and face-to-face interactions are essential [53, 69, 72]. As noted above, we followed these best practices, recruiting participants through community leaders, community events, and word of mouth from other participants [28, 31, 69]. Further, and based on conversations with community organizations and people at community events, it was very important to our participants that we were physically present and engaging with their community. We endeavored to do this by attending meetings, marches against violence, and other activities to be seen as worthy of having their participation in our research project. What we acknowledge is lost in representativeness by not using a specific sampling method, we gained in participant engagement and willingness to show up.
